# Focal Therapy: Patients, Interventions, and Outcomes—A Report from a Consensus Meeting

**DOI:** 10.1016/j.eururo.2014.09.018

**Published:** 2015-04

**Authors:** Ian A. Donaldson, Roberto Alonzi, Dean Barratt, Eric Barret, Viktor Berge, Simon Bott, David Bottomley, Scott Eggener, Behfar Ehdaie, Mark Emberton, Richard Hindley, Tom Leslie, Alec Miners, Neil McCartan, Caroline M. Moore, Peter Pinto, Thomas J. Polascik, Lucy Simmons, Jan van der Meulen, Arnauld Villers, Sarah Willis, Hashim U. Ahmed

**Affiliations:** a Division of Surgery and Interventional Science, University College London, London, UK; b Department of Urology, UCLH NHS Foundation Trust, London, UK; c Department of Clinical Oncology, Royal Marsden Hospital, London, UK; d Centre for Medical Image Computing, University College London, London, UK; e Department of Urology, L’Institut Mutualiste Montsouris, Paris, France; f Department of Urology, Oslo University Hospital, Oslo, Norway; g Department of Urology, Frimley Park Hospital NHS Foundation Trust, Frimley, UK; h Institute of Oncology, Leeds Teaching Hospitals NHS Trust, Leeds, UK; i Section of Urology, University of Chicago Medical Center, Chicago, IL, USA; j Department of Surgery, Memorial Sloan Kettering Cancer Center, New York, NY, USA; k Department of Urology, Hampshire Hospitals NHS Foundation Trust, Basingstoke, UK; l Department of Urology, Oxford University Hospitals NHS Trust, Oxford, UK; m Department of Health Services Research and Policy, London School of Hygiene and Tropical Medicine, London, UK; n Urologic Oncology Branch, National Cancer Institute, National Institutes of Health, Bethesda, MD, USA; o Division of Urology, Duke University Medical Center, Durham, NC, USA; p Department of Urology, Hôpital Huriez, CHRU Lille, Lille, France

**Keywords:** Prostatic neoplasms, Consensus development conference, Organ-sparing treatments

## Abstract

**Background:**

Focal therapy as a treatment option for localized prostate cancer (PCa) is an increasingly popular and rapidly evolving field.

**Objective:**

To gather expert opinion on patient selection, interventions, and meaningful outcome measures for focal therapy in clinical practice and trial design.

**Design, setting, and participants:**

Fifteen experts in focal therapy followed a modified two-stage RAND/University of California, Los Angeles (UCLA) Appropriateness Methodology process. All participants independently scored 246 statements prior to rescoring at a face-to-face meeting. The meeting occurred in June 2013 at the Royal Society of Medicine, London, supported by the Wellcome Trust and the UK Department of Health.

**Outcome measurements and statistical analysis:**

Agreement, disagreement, or uncertainty were calculated as the median panel score. Consensus was derived from the interpercentile range adjusted for symmetry level.

**Results and limitations:**

Of 246 statements, 154 (63%) reached consensus. Items of agreement included the following: patients with intermediate risk and patients with unifocal and multifocal PCa are eligible for focal treatment; magnetic resonance imaging–targeted or template-mapping biopsy should be used to plan treatment; planned treatment margins should be 5 mm from the known tumor; prostate volume or age should not be a primary determinant of eligibility; foci of indolent cancer can be left untreated when treating the dominant index lesion; histologic outcomes should be defined by targeted biopsy at 1 yr; residual disease in the treated area of ≤3 mm of Gleason 3 + 3 did not need further treatment; and focal retreatment rates of ≤20% should be considered clinically acceptable but subsequent whole-gland therapy deemed a failure of focal therapy. All statements are expert opinion and therefore constitute level 5 evidence and may not reflect wider clinical consensus.

**Conclusions:**

The landscape of PCa treatment is rapidly evolving with new treatment technologies. This consensus meeting provides guidance to clinicians on current expert thinking in the field of focal therapy.

**Patient summary:**

In this report we present expert opinion on patient selection, interventions, and meaningful outcomes for clinicians working in focal therapy for prostate cancer.

## Take Home Message

Focal therapy as an active treatment for prostate cancer is rapidly evolving. Expert opinion gathered in this report using robust consensus methodology captures current thinking and can help direct future research and clinical care.

## 1 Introduction

Focal therapy is gaining interest as a potential treatment for localized prostate cancer (PCa) [1]. In this rapidly evolving field, there is a need for robust trial designs to evaluate tissue-preserving strategies so that clinically meaningful outcomes can be presented to physicians and their patients. However, there has been much debate with respect to the ideal patient group, the type of intervention, and acceptable outcomes [2].

Researchers have been involved in a phased evaluation of focal therapy over the last 5 yr, culminating in a number of published studies summarized in a recent systematic review [1]. One of the next phases will involve greater targeting precision through the possible incorporation of accurate preoperative imaging—such as multiparametric magnetic resonance imaging (mp-MRI) to define the desired boundaries of ablation—at the time of the operative intervention.

An international consensus meeting of experts was convened to provide guidance on patient eligibility, interventions, and meaningful outcome measures for focal therapy in clinical practice and to assist in the development of a new focal therapy trial that will incorporate image fusion in the delivery of the ablative process. A number of consensus groups and panels reporting on focal therapy have used informal or formal consensus methodology [3–5]. Our current report used the formal RAND/University of California, Los Angeles (UCLA) Appropriateness Methodology as a two-stage consensus process.

## 2 Methods

The consensus panel consisted of 15 voting members, 1 independent chairperson with expertise in consensus methodology (J.vdM.), and 4 nonvoting observers (I.A.D., L.S., N.M., and S.W.). Members were selected for their expertise in focal therapy and clinical trials. Their background and experience are outlined in Table 1. The meeting was supported by a grant from the Wellcome Trust and the UK Department of Health to fund the evaluation of an MRI/ultrasound fusion device for targeted biopsy and focal therapy. Available funding limited the total number of participants.

The consensus process used the RAND/UCLA Appropriateness Methodology format [6]. The 237 items on which to derive consensus were formulated in two initial small-group rounds comprising I.A.D., C.M.M., J.vdM., S.W., A.M., and H.U.A., informed by current literature. Prior to a face-to-face meeting, all participants were asked to independently score these statements on a scale ranging from 1 (strongly disagree) to 9 (strongly agree).

**Fig. 1:**
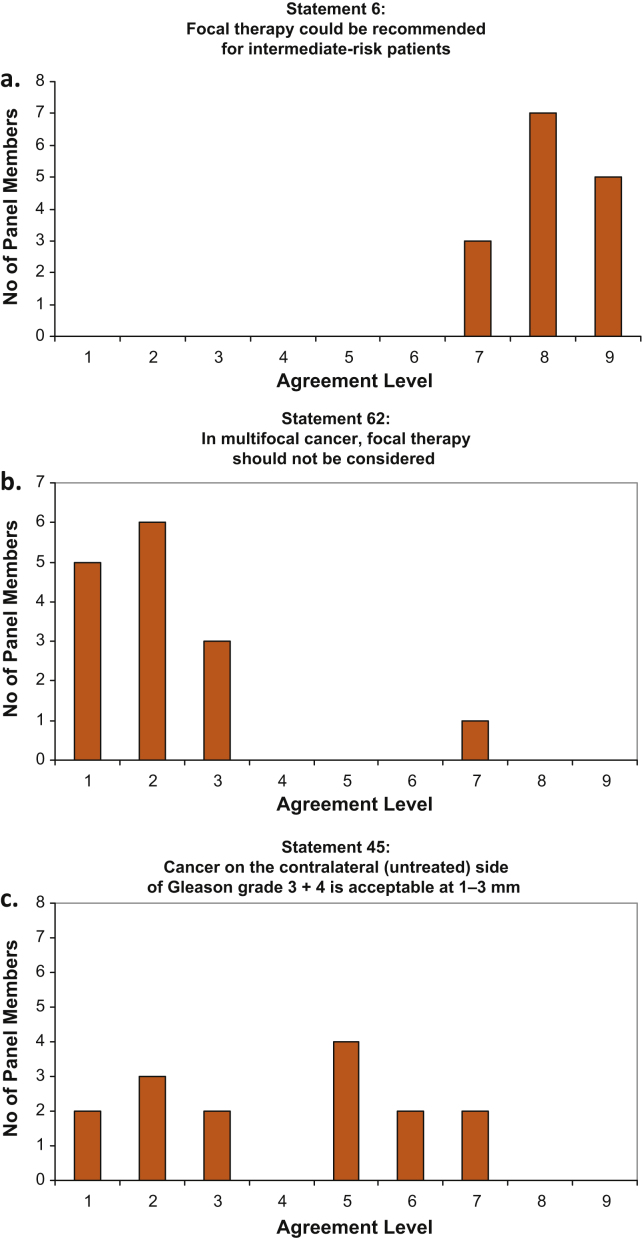
Examples of graphic results displayed to the panel. (a) Agree: median score: 8; interpercentile range adjusted for symmetry (IPRAS): 6.65. (b) Disagree: median score: 2; IPRAS: 6.65. (c) Uncertain: median score: 5; IPRAS: 1.65.

At the face-to-face meeting, the premeeting scores were displayed graphically (Fig. 1). After discussion, each panel member independently rescored all questions. Rewording and addition of statements were allowed if the original text was considered by the group to be ambiguous or not fully comprehensive.

After the meeting, agreement levels (disagree, uncertain, agree) for each statement were calculated as the median panel score. A median of 1–3 indicated disagreement with the statement; 4–6, uncertainty; and 7–9, agreement. The level of consensus (interpanel score variation) for each statement was calculated by the interpercentile range adjusted for symmetry (IPRAS) method [6]. An IPRAS score >0 indicates consensus among the group, with higher scores indicating a stronger consensus level. Only statements reaching agreement or disagreement can be included in these recommendations.

The results presented in this paper are expert opinion and therefore constitute level 5 evidence.

## 3 Results

All participants returned questionnaires prior to the meeting, and all attended the full day. From the 237 original statements, 17 additional statements were added, 46 were reworded, and 8 were removed during the panel discussion. The removed questions were considered to be unnecessary or outside the scope of this meeting.

As a result, 246 final statements were rescored at the face-to-face meeting. Consensus was reached for 154 statements (63%), indicating agreement for 85 and disagreement for 69. The full consensus document with final statements, agreement level, and IPRAS levels is included in Supplementary Table 1.

### 3.1 Definition of focal therapy

Various minimally invasive tissue ablation strategies exist for the treatment of localized PCa [1]. In clinical trials, ablation strategies have included hemi-ablation, so-called hockey-stick ablation (extended hemi-ablation), and quadrant ablation [2]. The panel agreed that focal therapy should be defined as ablation of the dominant or index lesion only. There was agreement that quadrant ablation is a possible focal therapy strategy, but with a lower level of consensus than lesion-only ablation.

Given that the ablative pattern of brachytherapy or cryotherapy differs from that of electroporation and high-intensity focused ultrasound, it was agreed that the morphology of the disease should guide the selection of the energy source to be used. If only one source of ablation is available, there was agreement that this situation would limit the type of focal therapy that could be delivered.

### 3.2 Patient selection

#### 3.2.1 Risk

There was agreement, with a high level of consensus, that based on current National Comprehensive Cancer Network classifications [7], focal therapy should be recommended for intermediate-risk patients. There was also agreement, with a lower level of consensus, for treating men with low-risk disease.

The shift in the attitude of the group over time from providing focal treatment to low-risk patients to now treating intermediate-risk patients was discussed. The shift was thought to be in part because of growing confidence in the technique and promising medium-term follow-up results [8,9]. The group recognized concerns about overdiagnosis and overtreatment [10–12] and agreed that providing focal therapy to men with well-characterized low-risk disease would represent overtreatment and that these men may be best served with active surveillance.

#### 3.2.2 Prostate volume

Acknowledging that some energy sources have limitations in their ability to treat some anatomic regions (eg, high-intensity focused ultrasound is limited to treating anterior lesions in small prostates only) while others do not, it was agreed that prostate volume should not be a primary determinant of eligibility for focal therapy.

#### 3.2.3 Age and life expectancy

It was agreed that age should not be a primary determinant of focal therapy, although the panel was uncertain about whether focal treatment should be recommended for patients <40 yr or >80 yr.

The panel was also asked to evaluate criteria other than age when selecting patients eligible for focal therapy. The panel agreed that patients with a World Health Organization performance status of 0 or 1 [13] should be recommended for focal treatment and that patients with a performance status of 3 or 4 should not be recommended. There was uncertainty about treating patients with a performance status of 2.

The group agreed that focal therapy was best suited to patients with a life expectancy of >10 yr and that this therapy should not be applied to patients with a life expectancy of ≤5 yr.

#### 3.2.4 Preintervention diagnostics

The panel agreed that a confirmatory tissue diagnosis of cancer should be available prior to performing focal therapy. There was a lack of agreement for providing focal treatment without a biopsy, given that the true positive predictive value of mp-MRI is yet to be fully quantified.

It was agreed that focal therapy can be performed in patients who have undergone an MRI-targeted prostate biopsy and in patients who have had a standard transrectal ultrasound (TRUS) biopsy in which the positive cores reflect, and are concordant with, a high-quality mp-MRI reported by an expert radiologist. When using an MRI-targeted strategy, the Standards of Reporting for MRI-targeted Biopsy Studies guidelines [14] should be followed.

#### 3.2.5 Disease visualization

For patients who have not had an mp-MRI because of lack of availability or physician preference, it was agreed that only a full transperineal template–mapping biopsy was sufficient to perform focal therapy [15]. The panel did not agree that the delivery of focal therapy can be based on only the information from a standard or extended TRUS biopsy without further imaging or template-mapping biopsies.

#### 3.2.6 Previous treatment

The panel agreed that focal therapy can be applied in patients who have already undergone one focal therapy and in patients who have had previous whole-gland treatment. In addition, focal therapy does not need to be limited to patients with a primary diagnosis of PCa and can be used in the setting of radiorecurrent disease [16] when the recurrent disease can be accurately localized.

### 3.3 Intervention

#### 3.3.1 Setting

The panel agreed that ideally, focal therapy should be delivered as a day case procedure, and it can be delivered in an office-based setting if the necessary equipment is in place.

#### 3.3.2 Multifocality

Specifically addressing the question of multifocal cancer, there was agreement that therapy should be targeted to the index lesion. There was no agreement about whether focal therapy should be targeted to all lesions. However, the panel agreed that multifocal cancer should not preclude focal therapy.

#### 3.3.3 Untreated disease

PCa is predominantly multifocal in the vast majority of men. The multifocal nature of PCa will mean that when using a focal therapy strategy to treat the primary PCa, some disease will be left untreated. As a result, the concept of index lesion ablation or ablation of one large, high-grade lesion has been proposed as a manner by which most men might undergo focal therapy [17]. At the heart of what threshold of disease clinicians are willing to leave untreated is the definition of what constitutes clinically significant disease.

The panel agreed that it was acceptable not to treat lesions of Gleason grade 3 + 3 up to a maximum cancer core length of 5 mm, although it has to be noted that the level of consensus was higher for not treating lesions with a smaller maximum cancer core length of 3 mm.

The panel agreed that it is not acceptable to leave untreated lesions with Gleason grade 3 + 4 with a maximum cancer core length of 5 mm or any 4 + 3 disease of any length. However, the panel did not reach consensus on whether lesions with Gleason grade 3 + 4 with a maximum cancer core length of 3 mm could be left untreated.

#### 3.3.4 Tumor volume

The panel did not agree on a maximum tumor volume beyond which focal therapy is deemed not suitable. Other factors needed to be considered, including the size of the prostate, the grade of the lesion, and the boundaries and morphologic characteristics of the lesion.

#### 3.3.5 Therapy planning

The panel agreed that ≤3 mm was an acceptable targeting error for software delivery of focal therapy to the center of the lesion. When performing focal therapy, an optimal circumferential margin for treatment was deemed to be 5 mm around a lesion that was seen on imaging. This is concordant with evidence that a targeting error in the order of 2–3 mm will achieve a positive hit rate of 90–95% of a 0.5-ml tumor and that MRI can underestimate tumor volume [18,19].

#### 3.3.6 Mode of treatment

In an attempt to define which treatment modality was preferred, multiple clinical scenarios were presented to the panel members. No clear preference emerged, with the discussion surrounding the idea that there is not enough evidence to support one treatment modality over another, and the best modality is the one that is available to the clinician and that the clinician has experience delivering.

### 3.4 Outcome

#### 3.4.1 Residual cancer

When using focal therapy to treat localized PCa, there is potential for cancer to remain within the intended treatment zone. The panel agreed that cancer in the treatment zone of Gleason grade 3 + 3 with a cancer core length ≤3 mm is clinically acceptable, but only if there is a decrease from the original cancer burden. In other words, the original cancer lesion should be of a higher grade or higher volume than the cancer that remains in the treatment field. Remaining lesions of Gleason grade 3 + 4 or 4 + 3 are never clinically acceptable, regardless of cancer core length.

#### 3.4.2 Post treatment biopsy

It was agreed that the optimal time for the first prostate biopsy after focal treatment is at 1 yr. A rising prostate-specific antigen level or suspicious areas on mp-MRI should also trigger biopsy. Patients who have had brachytherapy may need to wait 2 yr. The panel agreed that the biopsy should be performed in a targeted manner, as otherwise, previously untreated tissue could easily be inadvertently sampled. The panel remained uncertain about whether posttreatment biopsy should also routinely sample the untreated gland.

#### 3.4.3 Retreatment

The panel agreed that retreatment rates of ≤20% with focal therapy were clinically acceptable. There was agreement that any subsequent whole-gland therapy reflects a failure of focal therapy. A retreatment rate of ≤10% with whole-gland therapy was considered to be clinically acceptable.

## 4 Discussion

### 4.1 Summary of findings

The results of our consensus exercise following the RAND/UCLA Appropriateness Methodology provide guidance to clinicians who deliver focal therapy to patients with localized PCa. The results are also helpful to clinical researchers when planning studies evaluating the effectiveness of focal treatment compared with other treatment options.

### 4.2 Clinical and research implications

Our consensus panel agreed on a number of key points, which all represent a significant shift in the thinking about the potential role of focal treatment for patients with localized PCa. A first example is that the panel recommended focal therapy for men with intermediate-risk PCa. Also, the panel expressed the view that any energy modality could be used, provided that the capability of the ablative modality and the characteristics of the disease were taken into account when planning the treatment. Finally, it was agreed that focal treatment could be used for multifocal disease, secondary lesions ≤5 mm of Gleason 3 + 3 could be left untreated, and a reduction of grade or burden of disease within the treated area reflects treatment success. When selecting candidates for focal therapy, it is important that they be properly characterized. Reports of Gleason pattern 6 metastasizing exist [20] but have occurred in high-risk individuals who have undergone systemic therapy and would not be selected for focal treatment.

A number of uncertainties were identified and should be the topic of future research. The research questions include whether one therapeutic modality is better than another in terms of the ratio of side effects to cancer control, whether low-volume Gleason pattern 4 could be left untreated, and whether focal therapy compromises the application of a subsequent radical therapy in those men who might require it.

Whether longer-term comparative randomized studies can be delivered to answer these questions is an open question [21]. There remains significant skepticism on whether such a study is feasible in both ability to recruit and randomize and in terms of the size that would be required to demonstrate noninferiority of focal therapy compared with radical therapy, especially when survival is considered.

### 4.3 Limitations

Expert group meetings can be prone to significant bias since by their nature, they are composed of people who might have a vested interest in the field as a whole or in particular aspects of it. The presence of an independent chair, who had no personal interest in the results of the consensus exercise and who ensured that all panel members had the opportunity to contribute to the discussions and that no member dominated the discussions, reduced this bias. It is also important to note that the scoring method—also during the face-to-face meeting—was anonymous. We also acknowledge that there was a lack of expertise in imaging and pathology. Although this situation was decided a priori because the questions were about delivery of focal therapy, it may have affected our findings.

## 5 Conclusions

PCa treatment is evolving with the emergence of new ablative technologies and techniques. Guidance formulated using consensus methodology assists clinicians delivering focal therapy and helps inform future clinical trials and research programs. In this paper we have identified criteria for those men who may be suitable for focal therapy and disease states that could be treated, and we have outlined therapy-planning strategies and outcomes that might be legitimate and acceptable for clinical adoption if successfully met in prospective cohorts and trials.

  ***Author contributions:*** Ian A. Donaldson had full access to all the data in the study and takes responsibility for the integrity of the data and the accuracy of the data analysis.  

*Study concept and design:* Donaldson, Moore, Willis, Miners, Barratt, van der Meulen, Emberton, Ahmed.

*Acquisition of data:* Donaldson, Alonzi, Barret, Berge, Bott, Bottomley, Eggener, Ehdaie, Emberton, Hindley, Leslie, Moore, Pinto, Polascik, van der Meulen, Villers, Ahmed.

*Analysis and interpretation of data:* Donaldson, Willis.

*Drafting of the manuscript:* Donaldson.

*Critical revision of the manuscript for important intellectual content:* Donaldson, Alonzi, Barratt, Barret, Berge, Bott, Bottomley, Eggener, Ehdaie, Emberton, Hindley, Leslie, Moore, Pinto, Polascik, van der Meulen, Villers, Ahmed.

*Statistical analysis:* Donaldson, Willis.

*Obtaining funding:* None.

*Administrative, technical, or material support:* McCartan, Simmons.

*Supervision:* None.

*Other* (specify): None.  

***Financial disclosures:*** Ian A. Donaldson certifies that all conflicts of interest, including specific financial interests and relationships and affiliations relevant to the subject matter or materials discussed in the manuscript (eg, employment/affiliation, grants or funding, consultancies, honoraria, stock ownership or options, expert testimony, royalties, or patents filed, received, or pending), are the following: Ian A. Donaldson receives funding from the Department of Health, UK, and the Wellcome Trust. Dean Barratt is an investigator on projects funded by the Department of Health, UK; the Wellcome Trust; Prostate Cancer UK; the National Institute of Health Research, UK; and the UK Engineering and Physical Sciences Research Council. He has served as an unpaid consultant for SonaCare Medical LLC. Mark Emberton is a consultant to SonaCare Medical LLC, Steba Biotech, Sanofi Aventis, GlaxoSmithKline, Angiodynamics, and Sophiris. Neil McCartan has provided consultancy services for Steba Biotech and SonaCare Medical LLC. Caroline M. Moore receives funding from GlaxoSmithKline, the Wellcome Trust, Advanced Medical Diagnostics, and Sanofi and is a consultant for Steba Biotech. Thomas J. Polascik is a member of the board of the COLD registry and a consultant for Endocare. He is also an investigator for a clinical trial on Nanoknife (Angiodynamics). Sarah Willis receives funding from the Department of Health, UK, and the Wellcome Trust. Hashim U. Ahmed receives funding from the Wellcome Trust, National Institutes of Health Research–Health Technology Assessment program, the US National Institutes of Health–National Cancer Institute, Prostate Action, Medical Research Council (UK), and Prostate Cancer Research Centre; he also receives funding from SonaCare Medical LLC, Misonix, Oncura, and GE Healthcare for medical consultancy and is a consultant to Steba Biotech.  

***Funding/Support and role of the sponsor:*** The Department of Health, UK, and the Wellcome Trust had a role in the preparation and approval of the manuscript. This publication presents independent research supported by the Health Innovation Challenge Fund (HICF-T4–310), a parallel funding partnership between the Department of Health, UK, and the Wellcome Trust. The views expressed in this publication are those of the authors and not necessarily those of the Department of Health or the Wellcome Trust.

## Figures and Tables

**Table 1:** Consensus panel and focal therapy experience

Panel member	Specialty	Center	Most focal therapy experience	Other focal therapy experience
Hashim U. Ahmed	Urology	UCLH, UK	HIFU	Cryotherapy PDT Electroporation Brachytherapy
Roberto Alonzi	Oncology	Royal Marsden Hospital, UK	Brachytherapy	–
Eric Barret	Urology	L’Institut Mutualiste Montsouris, France	Cryotherapy	HIFU PDT Brachytherapy
Viktor Berge	Urology	Oslo University Hospital, Norway	HIFU	–
Simon Bott	Urology	Frimley Park Hospital, UK	HIFU	Cryotherapy PDT Brachytherapy
David Bottomley	Oncology	Leeds Teaching Hospitals, UK	Brachytherapy	–
Scott Eggener	Urology	University of Chicago Medical Centre, USA	Laser photothermal	–
Behfar Ehdaie	Urology	Memorial Sloan Kettering Cancer Center, USA	Cryotherapy	Electroporation PDT HIFU Brachytherapy
Mark Emberton	Urology	UCLH, UK	HIFU	Electroporation PDT Cryotherapy
Richard Hindley	Urology	Hampshire Hospitals, UK	HIFU	PDT Cryotherapy Electroporation
Tom Leslie	Urology	Oxford University Hospitals, UK	HIFU	PDT Brachytherapy
Caroline M. Moore	Urology	UCLH, UK	HIFU	PDT Brachytherapy
Peter Pinto	Urology	NIH, USA	Laser photothermal	HIFU Cryotherapy Brachytherapy
Thomas J. Polascik	Urology	Duke University Medical Centre, USA	Cryotherapy	Electroporation
Arnauld Villers	Urology	CHRU Lille, France	HIFU	PDT

HIFU = high-intensity focused ultrasound; NIH = National Institutes of Health; PDT = photodynamic therapy; UCLH = University College London Hospital.
