# Supplementary material for: Focal Therapy: Patients, Interventions, and Outcomes—A Report from a Consensus Meeting
Source: Eur Urol. 2015 Apr;67(4):771–7. doi: 10.1016/j.eururo.2014.09.018 (PMC4410301; doi:10.1016/j.eururo.2014.09.018)
Supplement: Supplementary file 1 [file mmc1.doc]

**Supplementary Table 1 – Consensus questionnaire with median result and IPRAS score**

PART 1 - Focal Therapy, Patients, Interventions and Outcomes

|  | **Question** | **Median** | **Statement Agreement Level** | **IPRAS** |
| --- | --- | --- | --- | --- |
|  | ***Focal Therapy Is:*** |  |  |  |
| 1 | The ablation of the lesion only | 8 | Agree | 6.6 |
| 2 | The ablation of one quadrant of the prostate | 8 | Agree | 5.1 |
| 3 | The ablation of one half of the prostate | 6 | Uncertain | 2.2 |
| 4 | Sub-total ablation of the prostate | 4 | Uncertain | -0.1 |
| *4 added* | Ablation of the Index Lesion | 8.5 | Agree | 6.6 |
|  |  |  |  |  |
|  | **PATIENT** |  |  |  |
|  | ***Focal Therapy Could be recommended for:*** |  |  |  |
| 5 | Low Risk Patients | 7 | Agree | 1.95 |
| 6 | Intermediate Risk Patients | 8 | Agree | 6.65 |
| 7 | Some High Risk Patients | 6 | Uncertain | 1.85 |
| 8 | High Risk Patients | 3 | Disagree | 2 |
|  |  |  |  |  |
|  | ***Maximum prostate volume for Focal Therapy:*** |  |  |  |
| 9 | Should be less than 20mls | 1 | Disagree | 6.6 |
| 10 | Should be 40mls | 2 | Disagree | 2.05 |
| 11 | Should be 60mls | 2 | Disagree | 3.45 |
| 12 | Should be 80mls | 2 | Disagree | 4.85 |
| 13 | Should be 100mls | 3 | Disagree | 1.95 |
| *13 added* | Prostate volume should not be a primary deterimant of eligibility for focal therapy | 9 | Agree | 8.35 |
|  |  |  |  |  |
|  | ***Focal Therapy should be considered in patients aged:*** |  |  |  |
| 14 | Less than 40 | 6 | Uncertain | 1.65 |
| 15 | 40 – 45 | 7 | Agree | 1.95 |
| 16 | 45 – 50 | 7 | Agree | 3.7 |
| 17 | 50 – 55 | 8 | Agree | 5.1 |
| 18 | 55 – 60 | 8 | Agree | 6.65 |
| 19 | 60 – 65 | 8 | Agree | 6.6 |
| 20 | 65 – 70 | 9 | Agree | 6.95 |
| 21 | 70 – 75 | 8 | Agree | 6.6 |
| 22 | 75 – 80 | 7 | Agree | 3.7 |
| 23 | 80 and over | 6 | Uncertain | 1.85 |
| *23 additional* | Age should not be a primary determinant for focal therapy | 9 | Agree | 8.35 |
|  |  |  |  |  |
|  | ***Patient performance status*** |  |  |  |
| 24 | Focal therapy should be considered for patients with a performance status of 0 | 9 | Agree | 8.35 |
| 25 | Focal therapy should be considered for patients with a performance status of 1 | 9 | Agree | 6.95 |
| 26 | Focal therapy should be considered for patients with a performance status of 2 | 6 | Uncertain | 1.4 |
| 27 | Focal therapy should be considered for patients with a performance status of 3 | 3 | Disagree | 1.95 |
| 28 | Focal therapy should be considered for patients with a performance status of 4 | 2 | Disagree | 5.2 |
|  |  |  |  |  |
|  | ***Focal Therapy should be applied to patients with a life expectancy of:*** |  |  |  |
| *24 added* | 5yrs | 3 | Disagree | 1.575 |
| *25 added* | 10yrs | 8 | Agree | 6.425 |
| *26 added* | 15yrs | 9 | Agree | 8.175 |
| *27 added* | 20yrs | 9 | Agree | 6.6 |
| *28 added* | 25yrs | 9 | Agree | 6.425 |
|  |  |  |  |  |
| 29 | A biopsy diagnosis of cancer is required in all patients | 9 | Agree | 6.95 |
| 30 | Focal therapy could be applied to pts who only have had standard TRUS biopsy with no MRI | 2 | Disagree | 6.6 |
| 31 | Focal therapy could be applied to pts who only have had extended TRUS biopsy with no MRI | 3 | Disagree | 1.6 |
| 32 | Focal therapy could be applied to pts who only have had transperineal mapping biopsy with no MRI | 8 | Agree | 4.9 |
| 33 | Focal therapy could be applied to pts who have undergone MRI-targeted biopsy | 9 | Agree | 6.6 |
| *33 added* | Focal therapy could be applied to patients with only a standard TRUS and with a concordent MRI | 7 | Agree | 3.35 |
|  |  |  |  |  |
| 34 | Focal therapy should be applied only in the primary setting | 2 | Disagree | 5.1 |
| 35 | Focal therapy can be applied to patients who have already had one focal therapy | 8 | Agree | 6.6 |
| 36 | Focal therapy can be applied to patients who have had previous whole gland treatment | 8 | Agree | 5.25 |
|  |  |  |  |  |
| ~~37~~ |  |  |  |  |
|  |  |  |  |  |
|  | **Untreated Cancer** |  |  |  |
|  | *Cancer on the contralateral (untreated) side of Gleason grade 3+3* |  |  |  |
| 38 | Is unacceptable | 2 | Disagree | 6.6 |
| 39 | Is acceptable at less than 1mm | 9 | Agree | 6.6 |
| 40 | Is acceptable at 1 - 3mm | 8 | Agree | 6.65 |
| 41 | Is acceptable at 3 - 5mm | 7 | Agree | 3.75 |
| 42 | Is acceptable at 5 – 10mm | 5 | Uncertain | 1.85 |
|  |  |  |  |  |
|  | *Cancer on the contralateral (untreated) side of Gleason grade 3+4* |  |  |  |
| 43 | Is unacceptable | 5 | Uncertain | 0.2 |
| 44 | Is acceptable at less than 1mm | 6 | Uncertain | 0.45 |
| 45 | Is acceptable at less than 1 - 3mm | 5 | Uncertain | 1.65 |
| 46 | Is acceptable at 3 - 5mm | 2 | Disagree | 3.15 |
| 47 | Is acceptable at 5 – 10mm | 2 | Disagree | 6.6 |
|  |  |  |  |  |
|  | *Cancer on the contralateral (untreated) side of Gleason grade 4+3* |  |  |  |
| 48 | Is unacceptable | 8 | Agree | 4.85 |
| 49 | Is acceptable at less than 1mm | 1 | Disagree | 5.2 |
| 50 | Is acceptable at 1 - 3mm | 1 | Disagree | 5.2 |
| 51 | Is acceptable at 3 - 5mm | 1 | Disagree | 6.6 |
| 52 | Is acceptable at 5 – 10mm | 1 | Disagree | 8.35 |
|  |  |  |  |  |
|  | **INTERVENTION** |  |  |  |
|  | ***Usability:*** |  |  |  |
| 53 | Focal therapy should be performed as a day case procedure | 9 | Agree | 6.6 |
| ~~54~~ |  |  |  |  |
|  |  |  |  |  |
| ~~55~~ |  |  |  |  |
| ~~56~~ |  |  |  |  |
| ~~57~~ |  |  |  |  |
|  | ***Availability:*** |  |  |  |
| 58 | Focal therapy should be performed in major prostate cancer centres only | 6 | Uncertain | -1.3 |
| ~~59~~ |  |  |  |  |
| ~~60~~ |  |  |  |  |
| 61 | Focal therapy could be performed in an office based setting | 7 | Agree | 2.55 |
|  |  |  |  |  |
|  | ***Lesion Multifocality:*** |  |  |  |
| 62 | In multifocal cancer, focal therapy should not be considered | 2 | Disagree | 6.65 |
| 63 | In multifocal cancer, focal therapy should be targeted to the largest lesion | 7 | Agree | 5.1 |
| 64 | In multifocal cancer, focal therapy should be targeted to all lesions | 4 | Uncertain | 1.85 |
| 65 | In multifocal cancer only whole gland therapy should be performed | 2 | Disagree | 5.1 |
|  |  |  |  |  |
|  | ***Disease Visualisation:*** |  |  |  |
| 66 | Focal therapy should be directed only to areas identified on TRUS with confirmatory biopsy | 2 | Disagree | 5.45 |
| 67 | Focal therapy should be directed only to areas identified on Doppler US with confirmatory biopsy | 2 | Disagree | 4.9 |
| 68 | Focal therapy should be directed only to areas identified only on HistoScan with confirmatory biopsy | 2 | Disagree | 3.5 |
| 69 | Focal therapy should be directed only to areas identified on mpMRI with confirmatory biopsy | 5 | Uncertain | -0.9 |
|  |  |  |  |  |
| 70 | Focal therapy should be directed to areas identified on histology when not seen on imaging | 7 | Agree | 5.1 |
|  |  |  |  |  |
|  | ***Tumour Volume:*** |  |  |  |
| 71added | The maximum tumour volume for focal therapy should be 1cc | 2 | Disagree | 6.6 |
| 71 | The maximum tumour volume for focal therapy should be 2cc | 2 | Disagree | 6.85 |
| 72 | The maximum tumour volume for focal therapy should be 5cc | 3 | Disagree | -0.1 |
| 73 | The maximum tumour volume for focal therapy should be 10cc | 7 | Agree | -1.9 |
| 74 | The maximum tumour volume for focal therapy should be 15cc | 2 | Disagree | 2.1 |
| 75 | The maximum tumour volume for focal therapy should be 20cc | 2 | Disagree | 2.1 |
| 76 | The maximum tumour volume for focal therapy can be greater than 20cc | 2 | Disagree | 6.6 |
|  |  |  |  |  |
|  | ***Treatment Registration:*** |  |  |  |
| 77 | An acceptable targeting error for therapy is 1mm | 8 | Agree | 6.6 |
| *77 added* | An acceptable targeting error for therapy is 3mm | 7 | Agree | 5.1 |
| 78 | An acceptable targeting error for therapy is 5mm | 4 | Uncertain | 1.85 |
| 79 | An acceptable targeting error for therapy is 10mm | 1 | Disagree | 6.6 |
|  |  |  |  |  |
|  | ***Treatment margins:*** |  |  |  |
| 80 | The treatment margin should be 1mm from the edge of the tumour | 2 | Disagree | 5.1 |
| 81 | The treatment margin should be 2 - 3mm from the edge of the tumour | 5 | Uncertain | -1.15 |
| 82 | The treatment margin should be 3 - 5mm from the edge of the tumour | 7 | Agree | 5.1 |
| 83 | The treatment margin should be 5 - 10mm from the edge of the tumour | 5 | Uncertain | -1.3 |
| 84 | The treatment margin should be 10 - 15mm from the edge of the tumour | 2 | Disagree | 5.1 |
| 85 | The treatment margin should be 15 - 20mm from the edge of the tumour | 1 | Disagree | 6.6 |
|  |  |  |  |  |
|  | **OUTCOME** |  |  |  |
|  | ***Residual cancer within the treated zone of grade 3+3*** |  |  |  |
| 86 | Is clinically unacceptable | 2 | Disagree | 6.85 |
| 87 | Is clinically acceptable to 1mm | 8 | Agree | 4.05 |
| 88 | Is clinically acceptable at 1 - 3mm | 7 | Agree | 3.75 |
| 89 | Is clinically acceptable at 3 - 5mm | 4 | Uncertain | 1.85 |
| 90 | Is clinically acceptable at 5 – 10mm | 2 | Disagree | 6.85 |
|  |  |  |  |  |
|  | ***Residual cancer within the treated zone of grade 3+4*** |  |  |  |
| 91 | Is clincally unacceptable | 4 | Uncertain | -2.55 |
| 92 | Is clinically acceptable to 1mm | 6 | Uncertain | 1.85 |
| 93 | Is clinically acceptable at 1 - 3mm | 2 | Disagree | 3.15 |
| 94 | Is clinically acceptable at 3 - 5mm | 2 | Disagree | 6.6 |
| 95 | Is clinically acceptable at 5 – 10mm | 1 | Disagree | 8.35 |
|  |  |  |  |  |
|  | ***Residual cancer within the treated zone of grade 4+3*** |  |  |  |
| 96 | Is clinically unacceptable | 9 | Agree | 6.6 |
| 97 | Is clinically acceptable to 1mm | 2 | Disagree | 4.85 |
| 98 | Is clincally acceptable at 1 - 3mm | 2 | Disagree | 6.6 |
| 99 | Is clinically acceptable at 3 - 5mm | 1 | Disagree | 6.95 |
| 100 | Is clinically acceptable at 5 – 10mm | 1 | Disagree | 8.35 |
|  |  |  |  |  |
|  | ***Biopsy Follow-up:*** |  |  |  |
| 101 | In clinical practice prostate biopsy should be performed as part of follow-up | 8 | Agree | 1.6 |
| 102 | In clinical practice prostate biopsy should be performed with a rising PSA post treatment | 8 | Agree | 5.1 |
| 103 | In clinical practice prostate biopsy should be performed on the basis of a suspicious mpMRI | 8 | Agree | 6.85 |
|  |  |  |  |  |
| 104 | In clinical practice the first prostate biopsy  should be performed at 2 weeks post procedure | 1 | Disagree | 6.6 |
| 105 | should be performed at 3 months post procedure | 1 | Disagree | 6.6 |
| 106 | should be performed at 6 months post procedure | 3 | Disagree | 0.2 |
| 107 | should be performed at 9 months post procedure | 2 | Disagree | 5.1 |
| 108 | should be performed at 1 year post procedure | 8 | Agree | 2.4 |
| 109 | should be performed at 18 months post procedure | 2 | Disagree | 5.1 |
| 110 | should be performed at 2 years post procedure | 2 | Disagree | 5.45 |
| 111 | should be performed at 3 years post procedure | 2 | Disagree | 5.1 |
| 112 | should be performed at 4 years post procedure | 2 | Disagree | 5.075 |
| 113 | should be performed at 5 years post procedure | 2 | Disagree | 6.85 |
|  |  |  |  |  |
| 114 | Prostate biopsy should sample the whole gland | 5 | Uncertain | 1.4 |
| 115 | Prostate biopsy should only sample the treated area | 5 | Uncertain | 0.25 |
| 116 | Prostate biopsy should only sample the untreated area | 4 | Uncertain | 1.6 |
|  |  |  |  |  |
| 117 | Prostate biopsy should be performed as a standard TRUS | 5 | Uncertain | 1.65 |
| 118 | Prostate biopsy should be performed in a 20 zone transperineal method | 4 | Uncertain | 1.6 |
| 119 | Prostate biopsy should be performed in a 5mm prostate mapping method | 4 | Uncertain | 1.6 |
| 120 | Prostate biopsy should be performed in a targeted method | 8 | Agree | 5.1 |
|  |  |  |  |  |
|  | ***Retreatment*** |  |  |  |
| 121 | Repeat focal treatment is acceptable | 8 | Agree | 5.1 |
| ~~122~~ |  | 7 | Agree | 5.1 |
|  |  |  |  |  |
| 123 | Retreatment rate of 5% is acceptable | 8 | Agree | 6.6 |
| 124 | Retreatment rate of 5 - 10% is acceptable | 8 | Agree | 6.6 |
| 125 | Retreatment rate of 10 - 15% is acceptable | 7 | Agree | 5.1 |
| 126 | Retreatment rate of 15 - 20% is acceptable | 7 | Agree | 2 |
| 127 | Retreatment rate of 20 - 25% is acceptable | 5 | Uncertain | 0.15 |
| 128 | Retreatment rate of greater than 25% is acceptable | 2 | Disagree | 5.1 |
|  |  |  |  |  |
| 129 | Any whole gland therapy is a failure of focal therapy | 7 | Agree | 0.25 |
|  |  |  |  |  |
| *130 added* | A retreatment rate of 5% with whole gland therapy is acceptable | 9 | Agree | 6.6 |
| *131 added* | 5 - 10% | 8 | Agree | 5.1 |
| *132 added* | 10 - 15% | 6 | Uncertain | -0.9 |
| *133 added* | 15 - 20% | 4 | Uncertain | 1.6 |
| *134 added* | 20 - 25% | 2.5 | Disagree | 3.15 |
| *135 added* | greater than 25% | 2 | Disagree | 6.6 |
|  | **PART 2 – Focal Therapy Energy Selection** |  |  |  |
|  | A **low risk** patient with an **anterior** tumour of **less than 0.5cc** |  |  |  |
| 1 | Electroporation | 6 | Uncertain | 1.95 |
| 2 | HIFU | 6 | Uncertain | -1.3 |
| 3 | Cryotherapy | 8 | Agree | 5.1 |
| 4 | PDT | 6 | Uncertain | 2.45 |
| 5 | Brachytherapy | 5 | Uncertain | 0.45 |
| 6 | Photothermal | 5 | Uncertain | 1.9 |
|  |  |  |  |  |
|  | A **low** **risk** patient with an **anterior** tumour of **0.5 to 1cc** |  |  |  |
| 7 | Electroporation | 7 | Agree | 1.85 |
| 8 | HIFU | 7 | Agree | 0.45 |
| 9 | Cryotherapy | 7 | Agree | 5.1 |
| 10 | PDT | 6 | Uncertain | 2.45 |
| 11 | Brachytherapy | 6 | Uncertain | 1.85 |
| 12 | Photothermal | 5 | Uncertain | 1.85 |
|  |  |  |  |  |
|  | A **low risk** patient with an **anterior** tumour of **1 – 5cc** |  |  |  |
| 13 | Electroporation | 5 | Uncertain | 2.1 |
| 14 | HIFU | 5 | Uncertain | 0.45 |
| 15 | Cryotherapy | 7 | Agree | 5.35 |
| 16 | PDT | 5 | Uncertain | 2.1 |
| 17 | Brachytherapy | 6 | Uncertain | 1.85 |
| 18 | Photothermal | 5 | Uncertain | 2.1 |
|  |  |  |  |  |
|  | A **low risk** patient with a **posterior** tumour of **less than 0.5cc** |  |  |  |
| 19 | Electroporation | 6 | Uncertain | 1.9 |
| 20 | HIFU | 8 | Agree | 5.1 |
| 21 | Cryotherapy | 8 | Agree | 5.1 |
| 22 | PDT | 7 | Agree | 3.75 |
| 23 | Brachytherapy | 7 | Agree | 3.7 |
| 24 | Photothermal | 6 | Uncertain | 1.65 |
|  |  |  |  |  |
|  | A **low** **risk** patient with a **posterior** tumour of **0.5 to 1cc** |  |  |  |
| 25 | Electroporation | 6 | Uncertain | 1.85 |
| 26 | HIFU | 8 | Agree | 6.85 |
| 27 | Cryotherapy | 8 | Agree | 5.1 |
| 28 | PDT | 7 | Agree | 3.7 |
| 29 | Brachytherapy | 7 | Agree | 3.7 |
| 30 | Photothermal | 6 | Uncertain | 1.65 |
|  |  |  |  |  |
|  | A **low risk** patient with a **posterior** tumour of **1 – 5cc** |  |  |  |
| 31 | Electroporation | 5 | Uncertain | 0.5 |
| 32 | HIFU | 8 | Agree | 5.1 |
| 33 | Cryotherapy | 7 | Agree | 5.1 |
| 34 | PDT | 6 | Uncertain | 3.6 |
| 35 | Brachytherapy | 7 | Agree | 3.35 |
| 36 | Photothermal | 5 | Uncertain | 0.2 |
|  |  |  |  |  |
|  | An **intermediate** risk patient with an **anterior** tumour of **less than 0.5cc** |  |  |  |
| 37 | Electroporation | 6 | Uncertain | 1.85 |
| 38 | HIFU | 7 | Agree | 0.25 |
| 39 | Cryotherapy | 7 | Agree | 3.7 |
| 40 | PDT | 6 | Uncertain | 2.1 |
| 41 | Brachytherapy | 7 | Agree | 1.6 |
| 42 | Photothermal | 5 | Uncertain | 2.15 |
|  |  |  |  |  |
|  | An **intermediate** risk patient with an **anterior** tumour of **0.5 to 1cc** |  |  |  |
| 43 | Electroporation | 6 | Uncertain | 1.85 |
| 44 | HIFU | 7 | Agree | 1.65 |
| 45 | Cryotherapy | 7 | Agree | 3.7 |
| 46 | PDT | 6 | Uncertain | 2.1 |
| 47 | Brachytherapy | 7 | Agree | 1.65 |
| 48 | Photothermal | 5 | Uncertain | 2.1 |
|  |  |  |  |  |
|  | An **intermediate** risk patient with an **anterior** tumour of **1 – 5cc** |  |  |  |
| 49 | Electroporation | 5 | Uncertain | 2.1 |
| 50 | HIFU | 6 | Uncertain | 1.85 |
| 51 | Cryotherapy | 7 | Agree | 3.4 |
| 52 | PDT | 5 | Uncertain | 2.1 |
| 53 | Brachytherapy | 7 | Agree | 1.85 |
| 54 | Photothermal | 5 | Uncertain | 2.35 |
|  |  |  |  |  |
|  | An **intermediate** risk patient with a **posterior** tumour of **less than 0.5cc** |  |  |  |
| 55 | Electroporation | 5 | Uncertain | 1.9 |
| 56 | HIFU | 8 | Agree | 6.65 |
| 57 | Cryotherapy | 8 | Agree | 5.45 |
| 58 | PDT | 7 | Agree | 1.65 |
| 59 | Brachytherapy | 7 | Agree | 1.95 |
| 60 | Photothermal | 5 | Uncertain | 1.9 |
|  |  |  |  |  |
|  | An **intermediate** risk patient with a **posterior** tumour of **0.5 to 1cc** |  |  |  |
| 61 | Electroporation | 5 | Uncertain | 1.9 |
| 62 | HIFU | 8 | Agree | 6.6 |
| 63 | Cryotherapy | 8 | Agree | 5.1 |
| 64 | PDT | 6 | Uncertain | 2 |
| 65 | Brachytherapy | 7 | Agree | 1.95 |
| 66 | Photothermal | 5 | Uncertain | 1.9 |
|  |  |  |  |  |
|  | An **intermediate** risk patient with a **posterior** tumour of **1 – 5cc** |  |  |  |
| 67 | Electroporation | 5 | Uncertain | 2.15 |
| 68 | HIFU | 8 | Agree | 3.35 |
| 69 | Cryotherapy | 7 | Agree | 3.35 |
| 70 | PDT | 6 | Uncertain | -0.075 |
| 71 | Brachytherapy | 7 | Agree | 2.2 |
| 72 | Photothermal | 5 | Uncertain | 2.1 |
|  |  |  |  |  |
|  | A **high risk** patient with an **anterior tumour** of less than 0.5cc |  |  |  |
| 73 | Electroporation | 5 | Uncertain | 1.85 |
| 74 | HIFU | 5 | Uncertain | 0.7 |
| 75 | Cryotherapy | 6 | Uncertain | 0.45 |
| 76 | PDT | 3 | Disagree | 1.65 |
| 77 | Brachytherapy | 5 | Uncertain | 0.325 |
| 78 | Photothermal | 4 | Uncertain | 1.6 |
|  |  |  |  |  |
|  | A **high risk** patient with an **anterior tumour** of 0.5 to 1cc |  |  |  |
| 79 | Electroporation | 4 | Uncertain | 1.65 |
| 80 | HIFU | 4 | Uncertain | 1.7 |
| 81 | Cryotherapy | 6 | Uncertain | 0.45 |
| 82 | PDT | 3 | Disagree | 1.95 |
| 83 | Brachytherapy | 4 | Uncertain | 1.7 |
| 84 | Photothermal | 4 | Uncertain | 1.95 |
|  |  |  |  |  |
|  | A **high risk** patient with an **anterior tumo**ur of 1 – 5cc |  |  |  |
| 85 | Electroporation | 3 | Disagree | 3.35 |
| 86 | HIFU | 3 | Disagree | 5.1 |
| 87 | Cryotherapy | 5 | Uncertain | -1.5 |
| 88 | PDT | 2 | Disagree | 4.9 |
| 89 | Brachytherapy | 3 | Disagree | 3.7 |
| 90 | Photothermal | 2 | Disagree | 3.7 |
|  |  |  |  |  |
|  | A **high risk** patient with a **posterior tumour** of less than 0.5cc |  |  |  |
| 91 | Electroporation | 4 | Uncertain | 1.65 |
| 92 | HIFU | 6 | Uncertain | 3.65 |
| 93 | Cryotherapy | 6 | Uncertain | 3.6 |
| 94 | PDT | 4 | Uncertain | 1.85 |
| 95 | Brachytherapy | 3 | Disagree | 0.2 |
| 96 | Photothermal | 3 | Disagree | 1.95 |
|  |  |  |  |  |
|  | A **high risk** patient with a **posterior tumour** of 0.5 to 1cc |  |  |  |
| 97 | Electroporation | 3 | Disagree | 3.7 |
| 98 | HIFU | 6 | Uncertain | 1.9 |
| 99 | Cryotherapy | 6 | Uncertain | 1.85 |
| 100 | PDT | 3 | Disagree | 3.7 |
| 101 | Brachytherapy | 2 | Disagree | 3.35 |
| 102 | Photothermal | 2 | Disagree | 3.35 |
|  |  |  |  |  |
|  | A **high risk** patient with a **posterior tumour** of 1 – 5cc |  |  |  |
| 103 | Electroporation | 2 | Disagree | 5.1 |
| 104 | HIFU | 3 | Disagree | 0.25 |
| 105 | Cryotherapy | 4 | Uncertain | -1.65 |
| 106 | PDT | 2 | Disagree | 5.25 |
| 107 | Brachytherapy | 2 | Disagree | 4.9 |
| 108 | Photothermal | 2 | Disagree | 5.25 |
|  |  |  |  |  |
